# Molecular analyses reveal close similarities between small cell carcinoma of the ovary, hypercalcemic type and atypical teratoid/rhabdoid tumor

**DOI:** 10.18632/oncotarget.6459

**Published:** 2015-12-04

**Authors:** Somayyeh Fahiminiya, Leora Witkowski, Javad Nadaf, Jian Carrot-Zhang, Catherine Goudie, Martin Hasselblatt, Pascal Johann, Marcel Kool, Ryan S. Lee, Tenzin Gayden, Charles W. M. Roberts, Jaclyn A. Biegel, Nada Jabado, Jacek Majewski, William D. Foulkes

**Affiliations:** ^1^ Department of Human Genetics, McGill University, Montreal, Quebec, Canada; ^2^ McGill University and Génome Québec Innovation Centre, Montreal, Quebec, Canada; ^3^ Department of Pediatrics, McGill University, Montreal, Quebec, Canada; ^4^ Institute of Neuropathology, University Hospital Münster, Münster, Germany; ^5^ Pediatric Hematology and Oncology, University Hospital Heidelberg, Heidelberg, Germany; ^6^ Division of Pediatric Neuro-Oncology, German Cancer Research Center DKFZ, Heidelberg, Germany; ^7^ German Cancer Consortium (DKTK), Core Center Heidelberg, Heidelberg, Germany; ^8^ Department of Pediatric Oncology, Dana-Farber Cancer Institute, Boston, Massachusetts, USA; ^9^ Department of Pediatrics, Keck School of Medicine of USC, Los Angeles, California, USA; ^10^ Department of Medical Genetics, Lady Davis Institute and Segal Cancer Centre, Jewish General Hospital, McGill University, Montreal, Quebec, Canada; ^11^ Department of Medical Genetics, Research Institute, McGill University Health Centre, Montreal, Quebec, Canada; ^12^ Current affiliation: Comprehensive Cancer Center and Department of Oncology, St. Jude Children's Research Hospital, Memphis, Tennessee, USA

**Keywords:** SCCOHT, ATRT, exome sequencing, SWI/SNF, methylation

## Abstract

Small cell carcinoma of the ovary, hypercalcemic type (SCCOHT) is the most common undifferentiated ovarian malignancy diagnosed in women under age 40. We and others recently determined that germline and/or somatic deleterious mutations in *SMARCA4* characterize SCCOHT. Alterations in this gene, or the related SWI/SNF chromatin remodeling gene *SMARCB1*, have been previously reported in atypical teratoid/rhabdoid tumors (ATRTs) and malignant rhabdoid tumors (MRTs). To further describe the somatic landscape of SCCOHT, we performed whole exome sequencing on 14 tumors and their matched normal tissues and compared their genomic alterations with those in ATRT and ovarian high grade serous carcinoma (HGSC). We confirmed that *SMARCA4* is the only recurrently mutated gene in SCCOHT, and show that recurrent allelic imbalance is observed exclusively on chromosome 19p, where *SMARCA4* resides. By comparing genomic alterations between SCCOHT, ATRT and HGSC, we demonstrate that SCCOHTs, like ATRTs, have a remarkably simple genome and harbor significantly fewer somatic protein-coding mutations and chromosomal alterations than HGSC. Furthermore, a comparison of global DNA methylation profiles of 45 SCCOHTs, 65 ATRTs, and 92 HGSCs demonstrates a strong epigenetic correlation between SCCOHT and ATRT. Our results further confirm that the genomic and epigenomic signatures of SCCOHT are more similar to those of ATRT than HGSC, supporting our previous hypothesis that SCCOHT is a rhabdoid tumor and should be renamed MRT of the ovary. Furthermore, we conclude that *SMARCA4* inactivation is the main cause of SCCOHT, and that new distinct therapeutic approaches should be developed to specifically target this devastating tumor.

## INTRODUCTION

Small cell carcinoma of the ovary, hypercalcemic type (SCCOHT) is the most common undifferentiated ovarian malignancy diagnosed in women under age 40, with a mean diagnosis age of 23.9 years [1]. It is an extremely aggressive tumor, with long-term survival rates of early stage diagnoses at 33% [2]. We and others recently discovered that SCCOHT is in fact a monogenic disease, where almost all cases are attributable to germline and/or somatic deleterious mutations in a single gene, *SMARCA4*, which is a key component of the SWI/SNF chromatin remodeling complexes [3-5]. These complexes utilize the energy of ATP hydrolysis to mobilize nucleosomes and remodel chromatin and have been found to promote transcriptional activation by inducing changes in DNA methylation patterns [6, 7].

Although SCCOHT is classified as a miscellaneous ovarian tumor by the World Health Organization [8], our previous publications describe its genetic and histological similarity to rhabdoid tumors [5, 9]. Rhabdoid tumors are pediatric soft tissue tumors that can manifest as either atypical teratoid/rhabdoid tumors (ATRTs) in the brain, or extra-cranial malignant rhabdoid tumors (MRTs) that most often develop in the kidney, but can arise in other tissues [10]. In 98% of cases, they are caused by inactivating mutations in *SMARCB1* (also known as *SNF5/INI1/BAF47*), another component of the SWI/SNF complex [6]. The other 2% are caused by deleterious mutations in *SMARCA4* (also known as *BRG1*), and when this is the case, the patients have been reported to have a worse prognosis [11]. While ATRTs and MRTs by definition arise in different tissues, gene expression profiling of ATRTs and MRTs has shown that they are molecularly similar tumors [10]. These tumors were originally thought to be variants of primitive neuroectodermal tumors (PNET) and Wilms tumor respectively, but were reclassified once genetic testing showed that they were distinct entities [9]. Likewise, our recent publications [5, 9] describe phenotypic and genetic similarities between SCCOHT, MRT, and ATRT, and propose that SCCOHT should be part of the rhabdoid tumor family and therefore be renamed MRT of the ovary (MRTO). Further similarities between MRT and SCCOHT include that hypercalcemia has been seen in both tumor types, with an incidence of approximately 30% in SCCOHT and 26% in patients with MRT [12]. Often, this serum hypercalcemia is due to increased parathyroid hormone production by the tumor, but it is still unclear whether the mechanism of hypercalcemia in these patients is related to the SWI/SNF mutations [12].

In contrast to SCCOHT, ovarian high grade serous carcinoma (HGSC) is the most common sub-type of ovarian cancer, accounting for 80-85% of all ovarian cancers, with a median age of diagnosis of 60 years. While the cell of origin of SCCOHT is still unknown, much work has been done on the characterization of HGSC; we therefore added HGSC to our comparison of SCCOHT and ATRT in an attempt to distinguish SCCOHT as a molecularly distinct entity from this common ovarian tumor.

In the present study, we further explore the landscape of somatic genetic and epigenetic alterations in SCCOHT and compare it to that of ATRTs and HGSC. Since SMARCA4 and SMARCB1 are two main components of the SWF/SNF complexes and are thought to modify DNA methylation in a similar manner, we hypothesized that they would show similarity in their global methylation profile. To our knowledge, no study has previously investigated the genome-wide methylation pattern of SCCOHT in comparison with other tumor types.

## RESULTS

### Somatic genomic alterations reveal SCCOHTs, like ATRTs, have “simple” genomes

To characterize the landscape of somatic alterations in SCCOHT, we performed whole exome sequencing (WES) on 14 formalin-fixed paraffin-embedded (FFPE) SCCOHT tumors and their matched normal tissues, and compared their overall genomic alterations with 14 ATRTs from our lab and 14 ovarian high-grade serous carcinomas (HGSCs) from TCGA.

**Figure 1:**
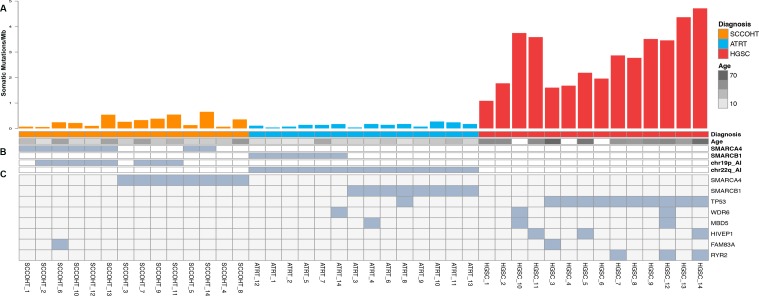
Results from genomic analysis of SCCOHT, ATRT, and HGSC **A**. Histograms show protein-coding somatic mutation rates for each sample in SCCOHT (orange), ATRT (blue) and HGSC (red). Age range of each patient is indicated below the histogram (age annotation was not available for HGSC_10, HGSC_4 and HGSC_6). **B**. Germline mutations identified in SMARCA4 and SMARCB1. **C**. Somatic mutations (substitutions and indels) and allelic imbalance (chr19p and chr22q for SCCOHT and ATRT, respectively). SCCOHT_4 and SCCOHT_8 had two somatic mutations each. The mutation in SCCOHT_3 was homozygous.

In line with previous studies, our WES analysis revealed *SMARCA4* and *SMARCB1* to be the only frequently mutated genes in SCCOHT and ATRTs, respectively (Figure 1, Table S1) [3-5, 13]. In contrast, HGSCs frequently showed mutations in *TP53*, consistent with the data published by TCGA [14]. This gene was somatically mutated in one ATRT (ATRT_8) and was not mutated in any SCCOHTs. Furthermore, no SCCOHTs had mutations in any of the eight other genes recurrently mutated in HGSC (*BRCA1, BRCA2, RB1, NF1, FAT3, CSMD3, GABRA6*, and *CDK12*) [14].

Consistent with the above results, Fisher's least significant difference (LSD) test revealed that the average number of somatic protein-coding mutations was significantly lower in SCCOHT (8.5 mutations) and ATRTs (4 mutations) than in HGSCs (74 mutations, p-values <0.0001 for both SCCOHT *vs* HGSC and for ATRT *vs* HGSC) (Figure S1). Moreover, our analysis also revealed fewer mutations per megabase (Mb) in the coding regions in SCCOHT and ATRTs (0.28 and 0.14 mutations/Mb, respectively) than in HGSCs (2.8 mutations/Mb) (Figure 1).

**Figure 2:**
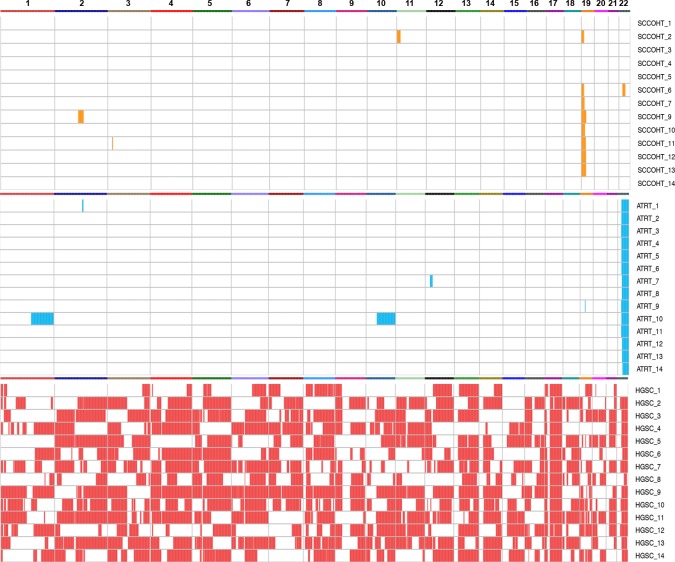
Genome-wide analysis of AI in SCCOHT (orange), ATRT (blue), and HGSC (red) Recurrent AI was observed only on chr19p and chr22q in SCCOHT and ATRTs, respectively, while many AI aberrations were detected across the genome of HGSCs.

To detect and compare recurrent somatic allelic imbalance (AI) in tumors, we analyzed WES data from each tumor type using ExomeAI (see methods) [15]. This analysis revealed remarkable genome-wide differences in the AI patterns of SCCOHT and ATRTs compared to those in HGSC (Figure 2). We found that SCCOHTs and ATRTs have very “simple” genomes and the only recurrent AI aberration identified was on chr19p surrounding *SMARCA4* (57% of cases), and chr22q surrounding *SMARCB1* (100% of cases), respectively (Figures 1 and 2). Regardless of the way in which the gene was altered, however, almost all SCCOHT and ATRT samples previously showed loss of the respective SMARCA4 or SMARCB1 protein (Table S1). In contrast to the quiescent SCCOHT and ATRT genomes, several abnormalities that included chromosomal arms or entire chromosomes were observed across the genome of HGSCs (Figure 2).

### The global DNA methylation patterns of SCCOHTs and ATRTs are strongly correlated

**Figure 3:**
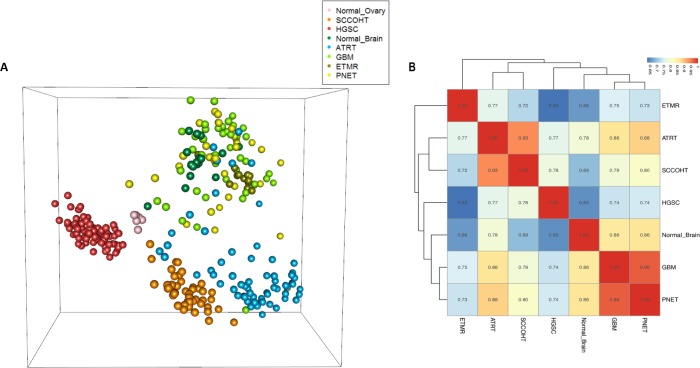
Methylation analyses of SCCOHT, ATRT, and HGSC compared to other samples **A**. Multidimensional scaling (MDS) analysis of methylation data. Methylation values of 10,000 most variable CpGs are projected into three dimensions. Each sphere represents a sample, with colors representing diagnostic groups as indicated in the legend. **B**. Hierarchical clustering of the methylation effect of diagnostic groups. The values used for clustering are segment-wise estimations of methylation group effects, as estimated by the model-based method (see methods). Pairwise correlation of the values (r) are shown. All pairs of correlations are highly significant (p-value < 10e-16). HGSC: High grade serous carcinoma; SCCOHT: Small cell carcinoma of the ovary, hypercalcemic type; ATRT: Atypical teratoid/rhabdoid tumors; GBM: Glioblastoma; ETMR: Embryonal tumor with multilayered rosettes; PNET: Primitive neuroectodermal tumor.

DNA methylation is a major regulator of gene expression and its alteration is frequently reported in tumorigenesis [16]. It is known that SWI/SNF complexes are involved in the establishment of DNA methylation patterns [7]. In order to characterize the genome-wide DNA methylation pattern of SCCOHT and to examine its similarity to ATRT and HGSC, we performed multidimensional scaling (MDS) analysis of methylation data using the 10,000 most variable CpGs. We found that the methylation pattern of SCCOHT was distinct from that of HGSC (Figure 3A). Similarly, ATRTs were clustered apart from other brain tumors, including glioblastomas, embryonal tumors with multilayered rosettes (ETMRs), and primitive neuroectodermal tumors (PNETs), although some degree of heterogeneity was present within the ATRT group. The latter is in line with a recent study that demonstrating that up to three subgroups may be detected in ATRTs [17].

We next quantified the degree to which methylation profiles of SCCOHT and ATRT are similar by applying a model-based analysis, which estimates the methylation effects between diagnostic groups, using a segment-wise approach (see methods). The analysis showed that SCCOHT has a higher Pearson correlation with ATRT (r = 0.93) than with HGSC (r = 0.78) (Figure 3B). Similar correlations (though of lesser magnitude) were obtained when applying a CpG-wise approach (Figure S3). Not surprisingly, all pairs of correlations are highly significant with *p*-values < 10e-16 (alternative hypothesis: true correlation is not equal to 0). This suggests that similar mechanisms, likely linked to chromatin remodeling by the SWI/SNF complexes, might contribute to similar methylation alterations in both groups (SCCOHT and ATRT).

## DISCUSSION

Understanding SCCOHT on a molecular level is crucial for determining the best treatment with which to combat the disease, as tissue of origin of a tumor is not always the best indicator of successful therapy.

Here we compared the genomic and epigenomic landscapes of SCCOHT, ATRT, and HGSC using WES and methylation analyses. Until now, several papers have discussed the similarities between SCCOHT and ATRT on genetic and histological levels [5, 9, 18], but this is the first study in which a comprehensive comparison has been made between these tumor types on genomic and epigenomic levels.

Using WES analysis, we were able to show the remarkable genomic similarities between SCCOHT and ATRT (Figures 1, 2, S2). Interestingly, while all ATRTs had LOH on chr22q (where *SMARCB1* is located) in addition to one *SMARCB1* mutation, only 8 of 14 SCCOHTs showed chr19p loss (where *SMARCA4* is located), with the remaining samples having only point mutations or small indels (see more details in Table S1). It is notable that one SCCOHT sample had only one mutation with no chr19p LOH. This sample did show loss of the SMARCA4 protein, but we were unable to find the second mutation by sequencing.

In addition to DNA sequencing analysis, analysis of the methylation data showed that SCCOHTs are considerably more similar to ATRTs than they are to HGSC (Figure 3). These genomic and epigenomic findings are mirrored by the clinical observations. The clinical presentation, histological appearance, and overall prognosis of SCCOHT differ from that of HGSC, in addition to loss of SMARCA4 in SCCOHT [19].

Perhaps the most valuable use of the results from our genomic and epigenomic analyses done here will be in forming new therapeutic strategies. Many case reports and studies of small cohorts of SCCOHT have described therapies combining surgical resection and adjuvant chemotherapy with many conflicting results. For SCCOHT, as with many cancers, stage at diagnosis seems to be the biggest factor in determining survival, with sometimes only surgery and radiotherapy needed for stage I patients [20]. For newly diagnosed SCCOHT, treatment is usually akin to that for the more common small cell lung cancer and ovarian germ cell tumors, where in addition to surgery, platinum-based therapy is used [21]. Other ovarian cancers, including HGSC, are treated similarly, by surgery with or without adjuvant chemotherapy, including platinums alone or in combination, depending on the stage at diagnosis.

While most patients do not survive a diagnosis of SCCOHT, intensive chemotherapy protocols have been occasionally found to result in durable responses, but mostly in early stage disease [22, 23]. For advanced stage disease, multiple combinations of almost all chemotherapy agents have been tried but with little success, as it has increasingly been shown that multi-modal therapy is required to effectively treat these patients [24]. This is similar to primary treatment for MRTs and ATRTs, where surgery has been shown to be beneficial with adjuvant chemotherapy and radiation therapy [25-27]. Additionally, high dose chemotherapy with stem cell rescue has been reported to be promising and has been incorporated in the primary treatment of ATRT [28, 29]. Similarly, some cases of SCCOHT have been treated with high-dose chemotherapy and stem cell rescue [30, 31].

Aside from current protocols, trials and *in vitro* studies in ATRT patients have resulted in some success. Due to the extensive research that has been done on the effects of SMARCB1 loss, many studies have tested the effect of targeting overexpressed proteins in ATRT. Alisertib (MLN8237), an Aurora K inhibitor, has shown early evidence of remarkable activity in the treatment of ATRT patients [32], while CDK, MEK, and EZH2 inhibitors have been shown to be effective in restricting tumor cell growth in rhabdoid tumor cell line and xenograft-based models [33-36]. In SCCOHT, on the other hand, no clinical trials have been conducted recently. While several recent papers show that SMARCA2, the ATPase counterpart of SMARCA4, is overexpressed in SMARCA4-deficient tumors and may be a good therapeutic target [37, 38], in the BIN-67 SCCOHT cell line, SMARCA2 is expressed at the mRNA level, but its protein is not detectable [5]. While BIN-67 is one of only two SCCOHT cell lines, it is indeed representative of SCCOHT tumors *in vivo,* as it was recently shown that loss of both SMARCA2 and SMARCA4 proteins is specific to SCCOHT [39]. Studies have been conducted in which other drugs and oncolytic viruses were tested in *in vitro* and *in vivo* models of SCCOHT [40,41], but thus far none have reached clinical trials.

Taken together, it is clear that not only is SCCOHT significantly more similar to ATRT than to HGSC on genetic and epigenetic levels, but that therapies used to treat ATRT seem to result in a better response in SCCOHT patients than those used to treat other types of ovarian cancer. Our methylation analysis results show that similar pathways may be dysregulated in SCCOHT and ATRT downstream of SMARCA4 or SMARCB1 loss. These results together with similarities between SCCOHT and MRT/ATRT on a histological and clinical level, demonstrate that management with multimodal therapy, including stem cell transplant, should be considered in the primary treatment of these devastating ovarian tumors. Furthermore, despite their distinct tissues of origin and ages at diagnosis, our data suggests that the loss of distinct SWI/SNF subunits leads to convergent biological features in ATRT and SCCOHT. This suggests an instructive role of the loss of subunits of this chromatin remodeling complex in shaping convergent cancer formation and dependencies.

## MATERIALS AND METHODS

### Patients and Tumor samples

To characterize the landscape of somatic alterations in SCCOHT, we performed WES on 14 formalin-fixed paraffin-embedded tumors and their matched normal tissues [42]. The mutation status of all 14 SCCOHT samples was previously published and all samples were previously seen by a gynecological pathologist to evaluate the histological appearance of SCCOHT [5]. To confirm the nature of SCCOHT tumors by loss of SMARCA4, immunohistochemistry on these tumors was done as previously described [5]. We then expanded the analysis by including 14 rhabdoid tumor samples (11 Brain, 1 kidney, 1 bladder, and 1 soft-tissue), carrying somatic or germline mutations in SMARCB1, from a previous study by Lee and colleagues [43], and 14 publically available HGSC samples from the TCGA consortium [44]. DNA methylation profiling was carried out on DNA extracted from tumor and normal ovarian tissue (45 SCCOHT and 6 normal ovary), and 110 previously published [45, 46] and 61 unpublished samples of tumor and normal brain tissue: 65 ATRTs, 31 PNETs, 12 ETMRs, 46 GBMs, and 17 normal brain samples. We also included methylation data from 12 TCGA HGSCs (Batch 409, Level 1 data downloaded from the TCGA data portal, https://tcga-data.nci.nih.gov/tcga/) and 80 HGSCs from a recent study by Patch et al. [47].

### Whole Exome Sequencing

Whole-exome library preparation, exon capture and sequencing were performed using our standard protocols at the McGill University and Génome Québec Innovation Centre as previously described by Witkowski et al. [5]. Briefly, DNA samples were extracted and visualized on an agarose gel. The Agilent SureSelect V4 and the Illumina Nextera Rapid-Capture Exome kits were used for whole exome capture as previously described [5]. All libraries were sequenced on either Illumina HiSeq 2000 or 2500 sequencer with paired-end 100-bp reads.

### Somatic genomic alterations analysis

Bioinformatics analysis of exome sequencing data was performed using our WES pipeline as previously described (Figure S2) [48, 49]. In brief, alignment of sequenced reads to the reference genome (hg19) was performed using BWA (v. 0.5.9) [50]. Subsequently, the Genome Analysis Toolkit (GATK) was used to do local realignment of reads around small insertions and deletions (indels) and to get coverage of consensus coding sequence (CCDS) bases (Table S1) [51]. A mean coverage of 76X (SCCOHT), 71X (ATRT) and 144X (HGSC) was obtained for all CCDS exons in each tumor type (for HGSCs, we also performed the analysis with 80X coverage which led to similar results; data not shown). Potential somatic substitutions, single nucleotide variants (SNVs) and indels, were called using Mutect (see https://confluence.broadinstitute.org/display/CGATools/MuTect for method) and IndelLocator (see https://confluence.broadinstitute.org/display/CGATools/Indelocator for methods) on the basis of BWA alignments and were then annotated with ANNOVAR [52]. To remove common variants and false positive calls, candidate somatic mutations were subjected to several filtering steps and eliminated if they fulfilled any one of the following criteria: (i) genomic position of variant covered by <10X, (ii) <5 reads support the alternative variant, (iii) variant has allelic ratio <5% for SNVs or <15% for indels, (iv) variant has allele frequency > 0.001 in our non-cancer (∼1000 exomes sequenced previously in our center) or ExAC databases, or (v) variant seen as homozygote in ExAC database. Finally, only the most likely damaging variants (nonsense, canonical splice-site, and missense mutations, and coding indels) were considered for further analysis.

To compare mutation rates of the three diagnostic groups (SCCOHT, ATRT, and HGSC), we used Fisher's least significant difference (LSD) test [53] with an alpha level of 0.05 and Bonferroni-adjusted P-values using the R package ‘agricolae’ (http://CRAN.R-project.org/package=agricolae).

AI analysis was performed on WES data using ExomeAI [15]. In brief, ExomeAI detects AI events across all samples by investigating the B allele frequency (BAF) profile of exomes. To do this, all heterozygous variants (BAF values of 0.05 to 0.95) are extracted from VCF files, and AI segments are called and summarized across all samples. In samples with low quality and quantity (e.g. FFPE tumors), AI-based methods produce more reliable calls, as inadequate genome-wide coverage consistency across tumor and matched normal samples can lead to false positive copy number calls [15].

### DNA Methylation analysis

Raw methylation intensities of 314 samples were imported and inspected for quality using the Bioconductor package Minfi [54]. Functional normalization was applied on all samples, where the first n (here n = 2) principal components of the internal control probes are used to adjust intensities for technical variations and batch effects [53, 55]. CpG sites were annotated using the IlluminaHumanMethylation450kanno.ilmn12.hg19 Bioconductor package, which is based on hg19, Illumina's version 1.2 annotations. Known SNP sites and CpGs on sex chromosomes were removed from further analysis. Similar to that described by Jaffe et al [56], we used a model-based method to estimate the methylation effect of all diagnostic groups, at both CpG and segment levels. Methylation effects of all diagnostic groups were estimated in comparison with normal ovary. For all model-based analyses, we used normalized beta values, as described above. More details of the method will be described elsewhere (J.N. et al, unpublished data). For MDS analysis, the most variable CpGs (n = 10,000) were used to calculate Euclidean distance between samples. Classical Multidimensional Scaling transformation was then applied to project the distances into 3 dimensions for visualization.

## SUPPLEMENTARY MATERIAL FIGURES AND TABLES


