# Supplementary material for: Molecular analyses reveal close similarities between small cell carcinoma of the ovary, hypercalcemic type and atypical teratoid/rhabdoid tumor
Source: Oncotarget. 2015 Dec 4;7(2):1732–40. doi: 10.18632/oncotarget.6459 (PMC4811493; doi:10.18632/oncotarget.6459)
Supplement: Supplementary file 1 [file oncotarget-07-1732-s001.pdf]

Molecular analyses reveal close similarities between small cell carcinoma of the ovary, hypercalcemic type and atypical teratoid/rhabdoid tumor

Supplementary Materials

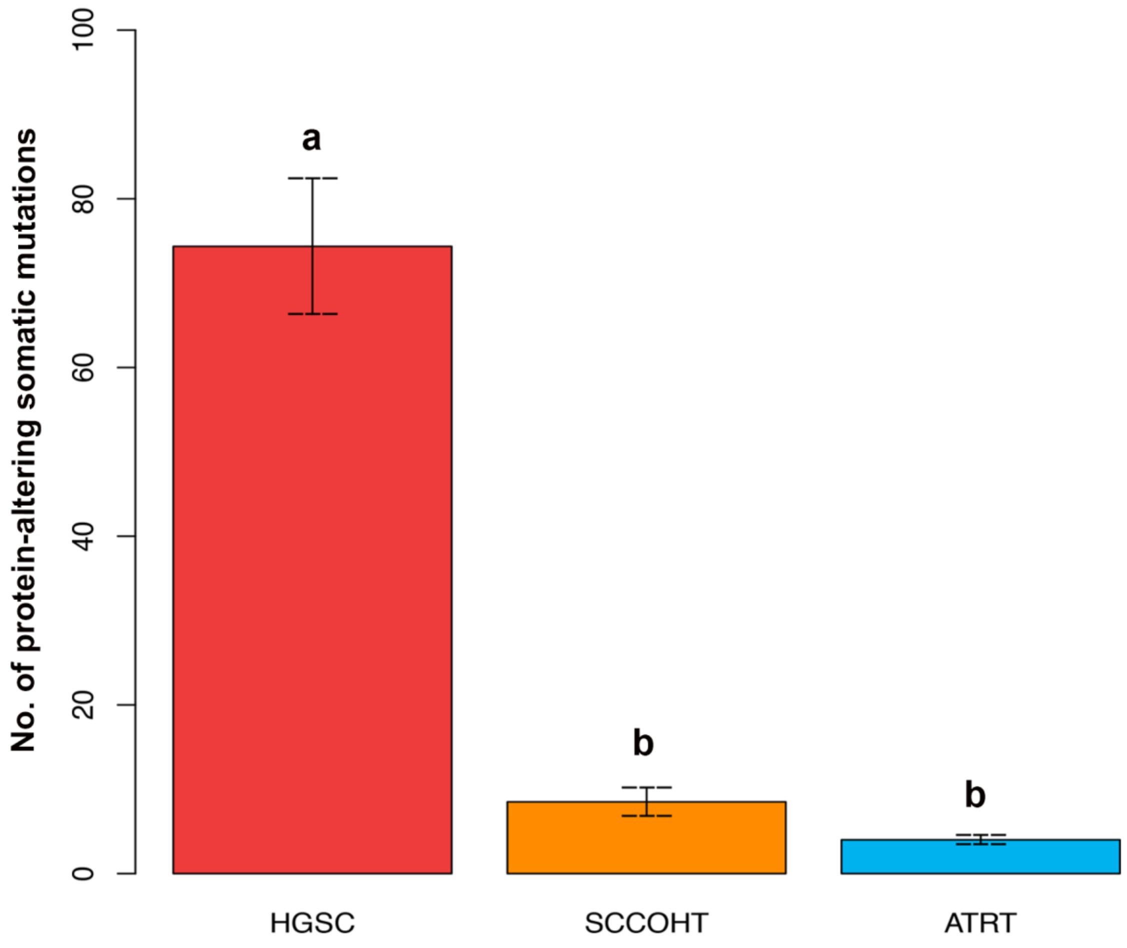

Figure S1: Comparison of mutation rates of the three tumor types (SCCOHT, ATRT, and HGSC) using Fisher's least significant difference (LSD) test. Error bars represent standard errors of means. Letter 'a' indicates significant difference between HGSC and SCCOHT and ATRT respectively (p-value <0.0001). Letter 'b' denotes no significant difference between SCCOHT and ATRT (significance level of 0.05). Number of samples used in each group is 14.

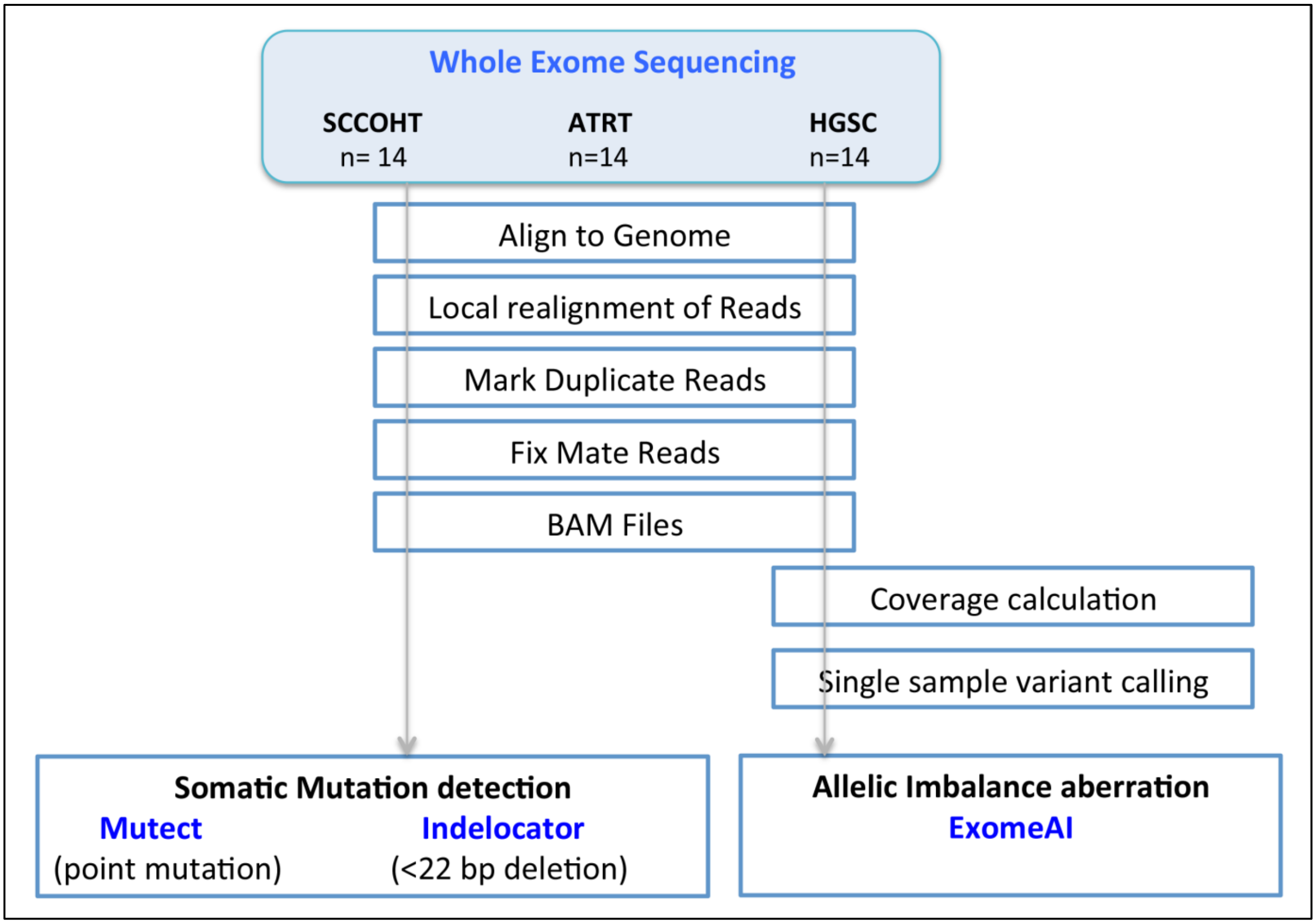

Figure S2: WES data analysis workflow: The WES raw data from SCCOHT, ATRT and HGSC were analyzed using our in-house WES pipeline [1, 2]. Downstream analysis of allelic imbalance (AI) and somatic mutations were performed using ExomeAI [3], Mutect and Indelocator, respectively.

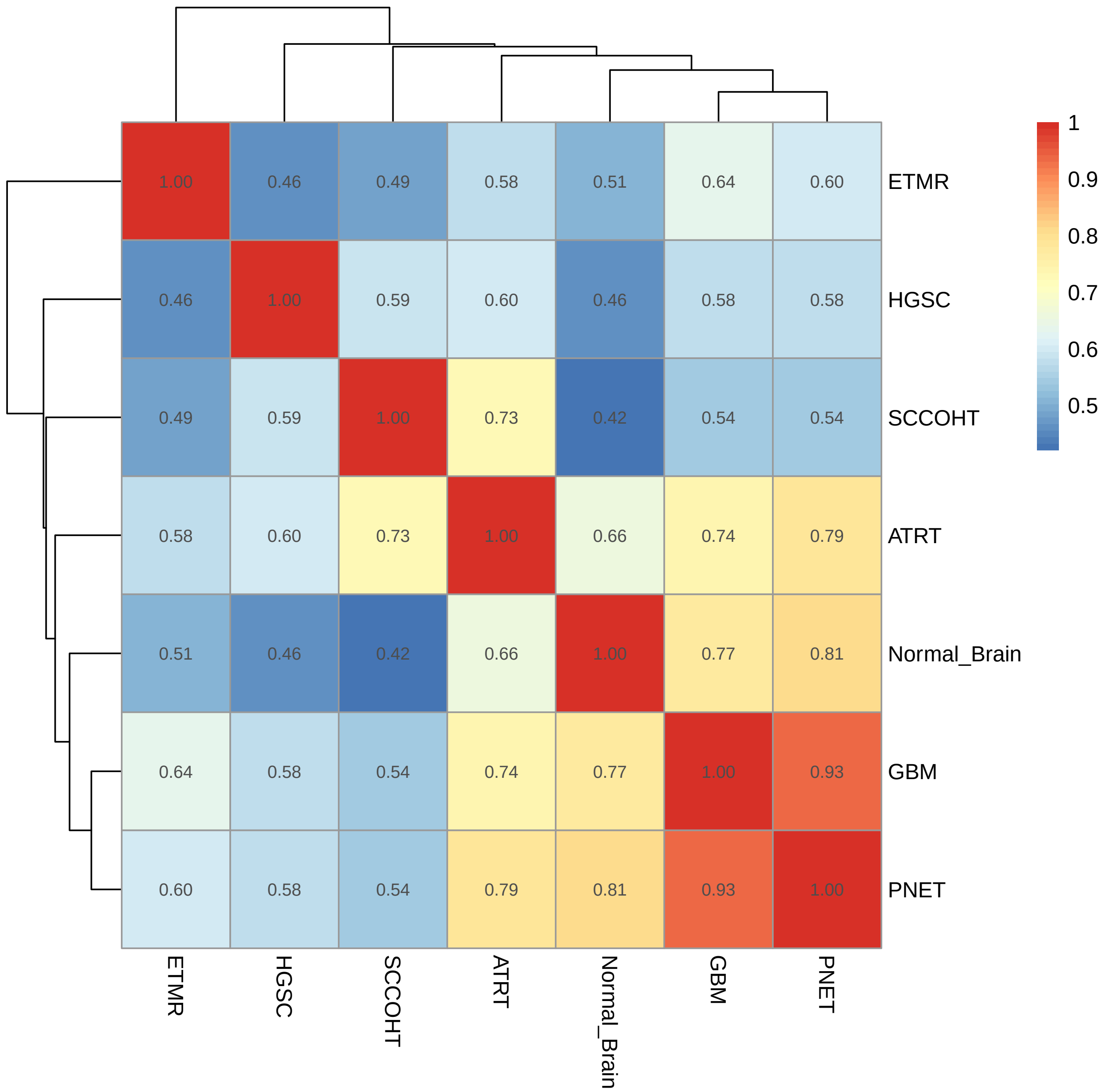

Figure S3: Hierarchical clustering of the methylation effect of diagnostic groups at CpG levels.

Table S1. Mutations found in SMARCA4 and SMARCB1 in SCCOHT and ATRT cases studied.

SCCOHT *SMARCA4* mutations			
**Sample ID**	**Mutation 1 (GL/S)**	**Mutation 2**	**Protein expression**
SCCOHT_1	c.3480_3481insG; p.Leu1161Alafs*15 (GL)	None	Loss
SCCOHT_2	c.1141 C>T; p.Arg381* (GL)	LOH	Loss
SCCOHT_3	c.1757_1757delA; p.Lys586Argfs*27 (S)	LOH	Loss
SCCOHT_4	c.2129_2129delC; p.Lys711Serfs*63 (S)	c.1378C>T;p.Gln460*	Loss
SCCOHT_5	c.3239G>A; p.Gly1080Asp (GL)	c.1326_1326delC; p.Ser442Argfs*59	Loss
SCCOHT_6	c.1224_1226delGCTinsAG; p.Leu409Glyfs*2 (GL)	LOH	Loss
SCCOHT_7	c.2275-1G>T (S)	LOH	Loss
SCCOHT_8	c.3531_3531delC; p.Trp1178Glyfs*38 (S)	c.4687_4687delG; p.Ile1564Serfs*32	Loss
SCCOHT_9	c.1663 C>T; p.Gln555* (GL)	LOH	Loss
SCCOHT_10	c.2617-3 C>G (GL)	LOH	Loss
SCCOHT_11	c.3496C>T; p.Gln1166* (S)	LOH	Loss
SCCOHT_12	c.4071+1 G>A (GL)	LOH	Loss
SCCOHT_13	c.3239G>A; p.Gly1080Asp (GL)	LOH	Retained
SCCOHT_14	c.643 C>T; p.Gln215* (GL)	c.1687_1700delAACCTCACGGAGCT; p.Asn563Glyfs*82	Loss
ATRT *SMARCB1* mutations			
**Sample ID**	**Mutation 1 (GL/S)**	**Mutation 2**	**Protein expression**
ATRT_1	c.472 C>T; p.Arg158* (GL)	LOH	Loss
ATRT_2	c.601 C>T; p.Arg201* (GL)	LOH	Loss
ATRT_3	c.778 C>T; p.Gln260* (S)	LOH	Loss
ATRT_4	c.552_553insGTGCTGGTCCCCA; p.Ile189fs*26 (S)	LOH	Loss
ATRT_5	c.93 G>C; p.Glu31Asp (GL)	LOH	Loss
ATRT_6	c.118 C>T; p.Arg40* (S)	LOH	Loss
ATRT_7	c.986+1 G>C (GL)	LOH	Loss
ATRT_8	c. 618G>A; p.Trp206* (S)	LOH	Loss
ATRT_9	c.157 C>T; p.Arg53* (S)	LOH	Loss
ATRT_10	c.118 C>T; p.Arg40* (S)	LOH	Loss
ATRT_11	c.1148_1148delC; p.Pro383Argfs*100 (S)	LOH	Loss
ATRT_12	c.94-2 A>G (GL)	LOH	Loss
ATRT_13	c.196_197insA; p.Ser67Valfs*4 (S)	LOH	Loss
ATRT_14	c.751_751delG; Asp251Thrfs*16 (GL)	LOH	Loss
GL, germline mutation; S, somatic mutation; LOH, Loss of heterozygosity. All samples showed loss of protein expression, aside from SCCOHT_13, discussed in Witkowski et al. 2014). Transcripts used are SMARCA4: NM_001128844 and SMARCB1: NM_003073			

Table S2. SCCOHT cases in this paper

Case	Methylation	Matched WES	SMARCA4 IHC	Reference
SCCOHT_1	No	Yes	Loss	[1] (NF5)
SCCOHT_2	Yes	Yes	Loss	[1] (UN7)
SCCOHT_3	No	Yes	Loss	[1] (NF9); [2]
SCCOHT_4	Yes	Yes	Loss	[1] (NF6)
SCCOHT_5	Yes	Yes	Loss	[1] (FA4)
SCCOHT_6	Yes	Yes	Loss	[1] (NF1)
SCCOHT_7	Yes	Yes	Loss	[1] (UN5)
SCCOHT_8	Yes	Yes	Loss	[1] (UN4)
SCCOHT_9	Yes	Yes	Loss	[1] (NF2)
SCCOHT_10	No	Yes	Loss	[1] (FA3)
SCCOHT_11	Yes	Yes	Loss	[1] (NF3)
SCCOHT_12	Yes	Yes	Loss	[1] (FA1)
SCCOHT_13	Yes	Yes	Retained	[1] (FA4)
SCCOHT_14	Yes	Yes	Loss	[1] (FA2)
SCCOHT_15	Yes	No	Loss	[1] (FA1)
SCCOHT_16	Yes	No	Loss	[1] (NF7)
SCCOHT_17	Yes	No	Loss	[1] (NF8)
SCCOHT_18	Yes	No	Loss	[1] (UN1)
SCCOHT_19	Yes	No	Loss	[1] (UN3)
SCCOHT_20	Yes	No	Loss	[1] (UN6)
SCCOHT_21	Yes	No	Loss	[1] (UN8)
SCCOHT_22	Yes	No	Retained	[1] (UN9)
SCCOHT_23	Yes	No	Loss	[1] (UN10)
SCCOHT_24	Yes	No	Loss	[1] (UN11)
SCCOHT_25	Yes	No	Loss	[1] (UN12)
SCCOHT_26	Yes	No	Loss	[1] (UN13)
SCCOHT_27	Yes	No	Loss	[1] (UN14)
SCCOHT_28	Yes	No	Loss	[1] (UN15)
SCCOHT_29	Yes	No	Loss	[1] (UN16)
SCCOHT_30	Yes	No	Loss	[5] (UN18)
SCCOHT_31	Yes	No	Loss	[5] (UN19)
SCCOHT_32	Yes	No	Loss	[5] (UN20)
SCCOHT_33	Yes	No	Loss	[5] (UN22)
SCCOHT_34	Yes	No	Loss	[5] (UN24)
SCCOHT_35	Yes	No	Loss	[5] (UN25)
SCCOHT_36	Yes	No	Loss	[5] (UN26)
SCCOHT_37	Yes	No	Loss	Unpublished
SCCOHT_38	Yes	No	Loss	Unpublished
SCCOHT_39	Yes	No	Loss	Unpublished
SCCOHT_40	Yes	No	Loss	Unpublished
SCCOHT_41	Yes	No	Loss	Unpublished
SCCOHT_43	Yes	No	Loss	Unpublished
SCCOHT_44	Yes	No	Loss	Unpublished
SCCOHT_45	Yes	No	Loss	Unpublished
SCCOHT_46	Yes	No	Loss	Unpublished
SCCOHT_47	Yes	No	Loss	Unpublished
SCCOHT_48	Yes	No	Loss	Unpublished
SCCOHT_49	Yes	No	Loss	Unpublished

References:

1. Witkowski L, Carrot-Zhang J, Albrecht S, Fahiminiya S, Hamel N, Tomiak E, Grynspan D, Saloustros E, Nadaf J, Rivera B *et al*: **Germline and somatic SMARCA4 mutations characterize small cell carcinoma of the ovary, hypercalcemic type**. *Nature Genetics* 2014, **46**(5):438-443.
2. Bailey S, Murray MJ, Witkowski L, Hook E, Hasselblatt M, Crawford R, Foulkes WD, Tischkowitz M, Nicholson JC: **Biallelic somatic SMARCA4 mutations in small cell carcinoma of the ovary, hypercalcemic type (SCCOHT)**. *Pediatric Blood & Cancer* 2015, **62**(4):728-730.
